# Supplementary material for: Effects of plyometric training on measures of physical fitness in racket sport athletes: a systematic review and meta-analysis
Source: PeerJ. 2023 Dec 15;11:e16638. doi: 10.7717/peerj.16638 (PMC10726777; doi:10.7717/peerj.16638)
Supplement: Supplemental Information 2 [file peerj-11-16638-s002.docx]

**Table S1. Detailed search strategy.**

**Search on 2 June 2023**

| **Databases** | **search strategy** | **Results** |
| --- | --- | --- |
| PubMed | ("plyometric training" [Title/Abstract] OR "ballistic training" [Title/Abstract] OR "jump training" [Title/Abstract] OR "plyometric exercise*" [Title/Abstract] OR "power training" [Title/Abstract] OR "stretch–shortening cycle" [Title/Abstract]) AND ("racquet sports" [Title/Abstract] OR "racket sports" [Title/Abstract] OR "racket players" [Title/Abstract] OR “badminton” [Title/Abstract] OR “squash” [Title/Abstract] OR “padel” [Title/Abstract] OR “tennis” [Title/Abstract] OR "table tennis"[Title/Abstract] OR "ping pong" [Title/Abstract] OR "racquetball" [Title/Abstract]) | 28 |
| Web of Science | (AB=(“plyometric training” OR "ballistic training" OR “jump training” OR “plyometric exercise*” OR “power training” OR “stretch–shortening cycle”)) AND AB=(” racquet sports” OR “racket sports” OR “racket players” OR “badminton” OR “squash” OR “padel” OR “tennis” OR “table tennis” OR “ping pong” OR “racquetball”) | 54 |
| (SPORTDicus) | AB ( “plyometric training” OR “ballistic training” OR “jump training” OR “plyometric exercise*” OR “power training” OR “stretch–shortening cycle” ) AND AB ( " racquet sports" OR "racket sports" OR "racket players" OR “badminton” OR “squash” OR “padel” OR “tennis” OR "table tennis" OR "ping pong" OR "racquetball" ) | 43 |
| Scopus | (TITLE-ABS-KEY("plyometric training" OR "ballistic training" OR "jump training" OR "plyometric exercise*" OR "power training" OR "stretch-shortening cycle") AND TITLE-ABS-KEY(" racquet sports" OR "racket sports" OR "racket players" OR badminton OR squash OR padel OR tennis OR "table tennis" OR "ping pong" OR "racquetball")) | 60 |
| Total |  | 185 |
