# Supplementary material for: Effects of plyometric training on measures of physical fitness in racket sport athletes: a systematic review and meta-analysis
Source: PeerJ. 2023 Dec 15;11:e16638. doi: 10.7717/peerj.16638 (PMC10726777; doi:10.7717/peerj.16638)
Supplement: Supplemental Information 3 [file peerj-11-16638-s003.docx]

| **Appendix 2** | **The data used for meta-analyses** | | | |  | | | | | |  | | |
| --- | --- | --- | --- | --- | --- | --- | --- | --- | --- | --- | --- | --- | --- |
| **Reference** | **Test** | **Experimental (pre-test)** | | | **Experimental (post-test)** | | | **Control**  **(pre-test)** | | | **Control**  **(post-test)** | | |
|  |  | **Mean** | **SD** | **n** | **Mean** | **SD** | **n** | **Mean** | **SD** | **n** | **Mean** | **SD** | **n** |
| Albayati et al., 2022 | Power (VJH) | 24.43 | 2.37 | 7 | 31.14 | 4.98 | 7 | 24.71 | 2.87 | 7 | 28 | 2.94 | 7 |
|  | Strength (handgrip) | 18.13 | 2.49 | 7 | 20.15 | 3.16 | 7 | 19.49 | 3.78 | 7 | 19.04 | 2.88 | 7 |
|  | Sprint speed (10 m) | 2.45 | 0.38 | 7 | 2.32 | 0.31 | 7 | 2.46 | 0.36 | 7 | 2.5 | 0.35 | 7 |
|  | Agility (t-test) | 10.89 | 0.25 | 7 | 10.2 | 0.73 | 7 | 10.73 | 1.27 | 7 | 10.74 | 0.73 | 7 |
|  | Reaction time | 0.65 | 0.09 | 7 | 0.66 | 0.09 | 7 | 0.66 | 0.04 | 7 | 0.65 | 0.09 | 7 |
| Behringer et al. 2013 | Strength (leg press) | 122.7 | 26 | 12 | 142.3 | 19.3 | 12 | 109.3 | 22.2 | 12 | 121.3 | 23.5 | 12 |
| Chou, 2022 | Power (VJH) | 46.75 | 12.56 | 8 | 54.47 | 15.83 | 8 | 45.62 | 11.94 | 8 | 52.24 | 10.54 | 8 |
|  | Sprint speed (30 m) | 4.55 | 0.56 | 8 | 4.17 | 0.83 | 8 | 4.69 | 0.94 | 8 | 4.54 | 0.51 | 8 |
|  | Agility (6 × 4 m shuttle run) | 7.45 | 0.36 | 8 | 6.58 | 0.73 | 8 | 7.56 | 0.62 | 8 | 6.94 | 0.34 | 8 |
| Chandra et al., 2023 | Agility (t-test) | 10.51 | 0.35 | 51 | 9.74 | 0.39 | 51 | 10.65 | 0.29 | 51 | 10.53 | 0.33 | 51 |
|  | Sprint speed (30 m) | 4.58 | 0.35 | 51 | 4.06 | 0.45 | 51 | 4.62 | 0.29 | 51 | 4.47 | 0.34 | 51 |
|  | Power (SBJ) | 181.17 | 6.05 | 51 | 178.30 | 5.97 | 51 | 183.02 | 3.89 | 51 | 183.88 | 3.91 | 51 |
| Heang et al., 2012 | Agility (Illinois test) | 22.46 | 2.92 | 19 | 20.86 | 2.58 | 19 | 23.64 | 2.91 | 23 | 22.99 | 2.66 | 23 |
| Haghighi et al., 2021 | Strength (Leg extension) | 54 | 17 | 10 | 67 | 23 | 9 | 59 | 15 | 10 | 61 | 16 | 10 |
| Kaabi et al., 2022 | Sprint speed (5 m) | 1.23 | 0.12 | 15 | 1.18 | 0.11 | 15 | 1.22 | 0.07 | 15 | 1.21 | 0.07 | 15 |
|  | Power (CMJ) | 35.06 | 1.78 | 15 | 37.25 | 1.77 | 15 | 34.63 | 2.3 | 15 | 35.02 | 2.5 | 15 |
|  | Strength (handgrip) | 32.49 | 2.8 | 15 | 36.25 | 3.24 | 15 | 31.77 | 2.04 | 15 | 31.99 | 2.16 | 15 |
|  | Agility (t-test) | 6.35 | 0.34 | 15 | 6.04 | 0.24 | 15 | 6.35 | 0.25 | 15 | 6.34 | 0.26 | 15 |
| Narang & Patil,2021 | Power (MBT) | 301.5 | 6.04 | 20 | 335.15 | 7.02 | 20 | 303.4 | 5 | 20 | 335.15 | 7.02 | 20 |
|  | Reaction time | 15.16 | 1.25 | 20 | 7.96 | 0.95 | 20 | 15.18 | 1.26 | 20 | 9.53 | 1.37 | 20 |
| Özmen & Aydoğmuş, 2017 | Agility (Illinois test) | 20.04 | 1.21 | 10 | 18.82 | 1.23 | 10 | 21.28 | 1.7 | 10 | 21.74 | 1.63 | 10 |
|  | Power (SJ) | 15.98 | 2.76 | 10 | 20.18 | 3.63 | 10 | 17.1 | 4.77 | 10 | 18.94 | 3.95 | 10 |
| Panda et al., 2022 | Power (VJH) | 46.20 | 7.22 | 30 | 48.26 | 7.79 | 30 | 46.70 | 4.13 | 30 | 47.4 | 3.97 | 30 |
|  | Sprint speed (30 m) | 4.95 | 0.29 | 30 | 4.76 | 0.27 | 30 | 4.56 | 0.15 | 30 | 4.57 | 0.22 | 30 |
|  | Agility (t-test) | 11.43 | 0.79 | 30 | 11.23 | 0.87 | 30 | 11.99 | 0.81 | 30 | 11.98 | 0.6 | 30 |
| Rathore et al. 2016 | Agility (Illinois test) | 18.71 | 0.69 | 20 | 16.58 | 0.44 | 20 | 18.55 | 0.71 | 20 | 17.13 | 0.88 | 20 |
| Salanikidis & Zafeiridis, 2008 EG1 | Power (DJ) | 13.6 | 4.4 | 16 | 18.3 | 6.1 | 16 | 12.7 | 3.8 | 8 | 14.9 | 3.6 | 8 |
|  | Strength (Fmax) | 1747 | 526 | 16 | 1886 | 580 | 16 | 872 | 334 | 8 | 923 | 346 | 8 |
|  | Sprint (12 m) | 2.47 | 0.2 | 16 | 2.44 | 0.22 | 16 | 2.46 | 0.19 | 8 | 2.42 | 0.18 | 8 |
|  | Reaction time | 221 | 35 | 16 | 162 | 32 | 16 | 230 | 30 | 8 | 196 | 23 | 8 |
| Salanikidis & Zafeiridis, 2008 EG2 | Power (DJ) | 13.8 | 5.5 | 16 | 16.9 | 4.8 | 16 | 12.7 | 3.8 | 8 | 14.9 | 3.6 | 8 |
|  | Strength (Fmax) | 1732 | 838 | 16 | 1874 | 883 | 16 | 872 | 334 | 8 | 923 | 346 | 8 |
|  | Sprint (12 m) | 2.47 | 0.19 | 16 | 2.41 | 0.19 | 16 | 2.46 | 0.19 | 8 | 2.42 | 0.18 | 8 |
|  | Reaction time | 222 | 71 | 16 | 158 | 36 | 16 | 230 | 30 | 8 | 196 | 23 | 8 |
| Zaferanieh et al., 2021 | Power (SJT) | 50.1 | 13.2 | 10 | 51.1 | 10.4 | 10 | 47.7 | 5 | 10 | 47.8 | 5.3 | 10 |
|  | Agility (t-test) | 9.89 | 0.7 | 10 | 10.94 | 0.66 | 10 | 10.63 | 0.74 | 10 | 10.95 | 0.3 | 10 |
|  | Reaction time | 169 | 16 | 10 | 175 | 26 | 10 | 167 | 18 | 10 | 171 | 23 | 10 |
|  | Strength (handgrip) | 110 | 19 | 10 | 118 | 16 | 10 | 117 | 25 | 10 | 121 | 24 | 10 |

Note: VJH, vertical jump height; CMJ, countermovement jump; EG, experimental group, MBT, medicine ball throw; DJ, drop jump; SJT, Sargent jump test; Fmax, maximum isometric force (knee extension); SBJ, standing broad jump; SJ, squat jump.
